# Combination Therapy With Sintilimab and Anlotinib for Advanced Undifferentiated Pleomorphic Sarcoma: A Case Report and Literature Review

**DOI:** 10.1002/ccr3.73006

**Published:** 2026-06-22

**Authors:** Yan Chen, Ronghua Chu

**Affiliations:** ^1^ Ningbo Beilun District Traditional Chinese Medicine Hospital Ningbo China

**Keywords:** anlotinib, pulmonary metastasis, sintilimab, undifferentiated pleomorphic sarcoma

## Abstract

This report describes the case of a 73‐year‐old woman with advanced undifferentiated pleomorphic sarcoma (UPS) who was intolerant to chemotherapy. Following amputation, she received sintilimab plus anlotinib and achieved a partial response with 76.6% tumor reduction. This combination may be a potential option for chemotherapy‐ineligible advanced UPS, warranting further investigation.

## Key Clinical Message

For older patients with advanced undifferentiated pleomorphic sarcoma who are intolerant to chemotherapy, the combination of sintilimab and anlotinib may offer a promising salvage treatment option. Furthermore, integrating systemic therapy with timely local control (e.g., amputation) can lead to significant tumor regression and prolonged disease control.

## 1 Introduction

Soft tissue sarcoma (STS) is a mesenchymal tissue‐derived malignant tumor. It is characterized by marked heterogeneity and rarity, accounting for approximately 1% of all human malignancies and encompassing more than 50 distinct histological subtypes [1, 2]. Undifferentiated pleomorphic sarcoma (UPS), formerly referred to as malignant fibrous histiocytoma (MFH), accounts for 10%–20% of STS cases and is particularly prevalent among older adults, with a peak incidence between the ages of 50 and 70 years [3, 4]. UPS is the most common histological subtype of STS involving the trunk and extremities [5]. Approximately 50%–70% of UPS patients are associated with a 5‐year overall survival (OS) rate [2]. UPS management poses a considerable clinical challenge owing to its propensity for local recurrence and the substantial risk of distant metastasis [6]. Despite surgical resection achieving local control of the primary tumor, reported local recurrence rates range from 13% to 52%, while distant metastases occur in 31%–35% of cases [7, 8]. Among patients with metastatic disease, the lung is the most commonly affected organ (76.9%), and these patients often present with poorer clinical outcomes at diagnosis [9, 10].

Doxorubicin‐based chemotherapy remains the standard systemic treatment for advanced UPS. However, molecularly targeted agents and immune checkpoint inhibitors have demonstrated superior efficacy compared with standard therapies across multiple malignancies [4, 5]. In this report, we describe the case of a 73‐year‐old female patient with high‐grade UPS of the lower left leg. Following surgical resection with positive margins, intolerance to first‐line chemotherapy, and a one‐year treatment gap, the tumor rapidly recurred, became ulcerated and infected, and later metastasized to the lungs. In response to this clinical course, we employed a combination of sintilimab (an anti‐programmed cell death protein‐1 antibody) and anlotinib (a tyrosine kinase inhibitor), together with amputation for local disease control. After treatment, the patient's pulmonary metastases revealed significant shrinkage, achieving a partial response (PR). These results provide novel insights and serve as a valuable clinical reference for the management of refractory advanced STS.

## 2 Case Examination

All patient information presented in this case report was anonymized; therefore, ethical approval was not required. Written informed consent was procured from the patient prior to the start of treatment.

This case involves a 73‐year‐old female patient of Asian descent. In October 2022, she underwent excision of a mass in the left lower leg at an external hospital after its initial detection, and postoperative pathological analysis indicated high‐grade STS. In November 2022, she was moved to a higher‐level hospital for wide local excision of the left lower limb soft tissue malignancy, combined with tendon repair and artificial skin grafting. Postoperative pathological examination revealed a malignant spindle cell tumor with positive surgical margins, consistent with UPS. Immunohistochemistry results were as follows: CK(pan)(−), SATB2(−), S‐100(−), SMA(−), Desmin(+), CD34(−), P63(−), CD68(−), CD56(+), Ki‐67(+30%), P16(+), P53(−), H3K27Me3(+), CD31(−), ERG(+), Myogenin(−), ALK‐D5F3‐Neg(−). Based on these findings, a final diagnosis of spindle cell UPS of the left lower leg was established. No postoperative adjuvant chemotherapy or radiotherapy was administered.

In April 2024, the patient visited the hospital with symptoms involving left lower leg pain. Magnetic resonance imaging (MRI) of the left lower leg performed at an external hospital revealed a soft tissue mass located in the anterior aspect of the distal left tibia with associated bone involvement, findings consistent with malignant tumor recurrence in the context of her clinical history. Positron emission tomography (PET)/computed tomography (CT) further confirmed recurrence of the soft tissue tumor in the anterior aspect of the distal left lower leg with tibial invasion, along with a metastatic lesion in the posterior segment of the right upper lobe (approximately 12 × 10 mm). Although repeat local surgery of the left lower leg was recommended at the external hospital, the patient declined surgical intervention. She subsequently initiated single‐agent chemotherapy with epirubicin (80 mg administered as an intravenous infusion on day 1 of a 3‐week cycle) on May 18, 2024. However, treatment was discontinued owing to severe, intolerable adverse reactions, including nausea, vomiting, abdominal pain, and dizziness. The patient received no further treatment during the following year.

In July 2025, the patient experienced recurrent pain in the left lower leg, which progressively worsened and was associated with restricted mobility. Specialist examination revealed an ulcer measuring approximately 7 × 7 cm in the anterior tibial region of the left lower leg, characterized by brown necrotic tissue, yellowish purulent foul‐smelling exudate, erythematous margins, depth reaching the bone surface, and marked tenderness on palpation. The patient was subsequently admitted to our hospital for further treatment.

## 3 Methods

**FIGURE 1:**
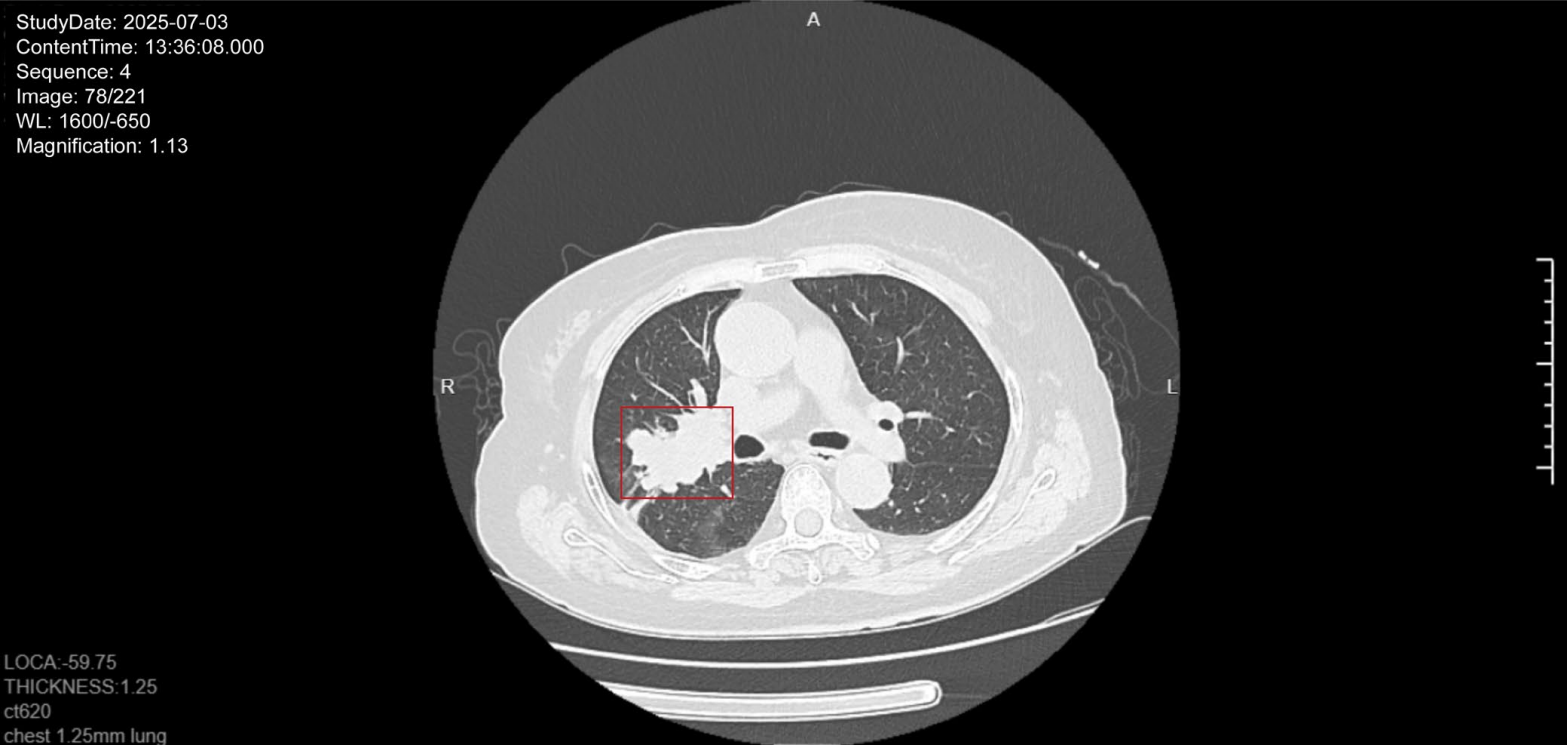
Chest computed tomography performed after admission revealed an irregular mass (approximately 47 × 30 mm) adjacent to the hilum of the right upper lobe.

After admission, the patient was administered ceftazidime (2 g bid intravenous infusion) for infection control and sustained‐release morphine tablets (10 mg q12h orally) for analgesia, with relevant investigations completed. A chest CT scan performed on July 3, 2025, revealed an irregular mass (approximately 47 × 30 mm) adjacent to the right upper lobe hilum (Figure 1). Following confirmation of advanced‐stage pulmonary metastasis, a combined immunotherapy and targeted therapy regimen was initiated on July 7, 2025, consisting of sintilimab (200 mg administered as intravenous infusion on day 1 of a 3‐week cycle) and anlotinib (10 mg orally on days 1–14 of each 3‐week cycle).

Owing to the severe ulceration of the left lower limb, suboptimal response to antibiotic therapy, significant tumor burden, and poor general condition, an orthopedic consultation was obtained. The patient subsequently underwent a left lower leg amputation under spinal anesthesia on July 14, 2025. Postoperative pathological examination confirmed the presence of a necrotic soft tissue tumor. The original treatment regimen was resumed after surgery, and combined therapy was administered on August 22, September 18, October 16, and November 13, 2025. As of the submission date, the patient had completed a total of five cycles of combination therapy.

## 4 Outcome and Follow‐Up

**FIGURE 2:**
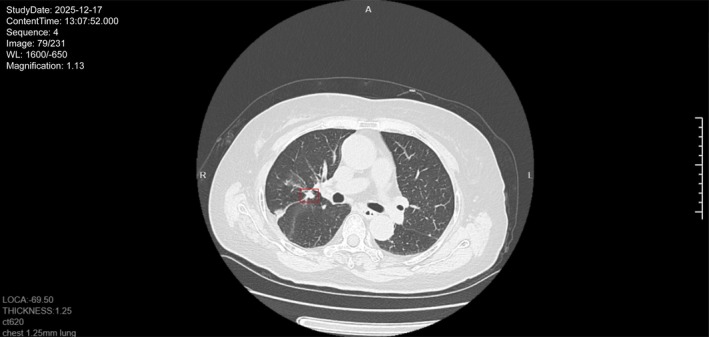
Follow‐up chest computed tomography performed on December 17, 2025, showed continued improvement, with further reduction of the right lung mass to 11 × 7 mm.

Tumor response was assessed according to RECIST version 1.1. The target lesion was defined as a mass adjacent to the right upper lobe hilum (baseline size, 47 × 30 mm). At the first follow‐up (September 17, 2025), the lesion had decreased to 18 × 12 mm, representing a 61.7% reduction in the sum of longest diameters and meeting the criteria for partial response (PR). At the second follow‐up assessment (December 17, 2025), the lesion had further decreased to 11 × 7 mm, corresponding to a 76.6% reduction from baseline, with PR maintained (Figure 2). No new lesions or definite progression of non‐target lesions were observed. During the five cycles of sintilimab plus anlotinib therapy, no grade 3 or 4 treatment‐related adverse events were reported. The patient experienced grade 1 fatigue, grade 1 anorexia, and grade 2 hyperlipidaemia, all of which resolved spontaneously without dose modification or treatment interruption. At the time of manuscript submission, the patient reported occasional pain in the affected limb, and the surgical incision on the left calf had healed well.

## 5 Discussion

UPS is a rare and highly aggressive mesenchymal tumor, the diagnosis of which requires confirmation via biopsy and comprehensive histological assessment by an STS pathologist [11, 12]. Morphologically, UPS is characterized by marked cellular pleomorphism, spindle‐shaped tumor cells, pronounced nuclear atypia and hyperchromasia, absence of lineage‐specific differentiation, and a disorganized or storiform cellular arrangement, with the vast majority being high‐grade tumors [13]. UPS diagnosis is exclusive and requires the exclusion of key differential diagnoses, including poorly differentiated carcinoma, melanoma, dedifferentiated liposarcoma, pleomorphic liposarcoma, and pleomorphic leiomyosarcoma [14].

The pathogenesis of UPS remains incompletely elucidated [15]. Research suggests that the core mechanism involves high genomic instability, manifested as complex chromosomal numerical and structural abnormalities, along with functional loss of key tumor suppressor pathways, such as TP53 and RB1 [16]. Within this molecular context, several oncogenic signaling pathways, including the PI3K/Akt/mTOR and RAS/MAPK pathways, are abnormally activated. In addition, dysregulation of the Hippo pathway effectors, particularly YAP/TAZ, collectively forms a pro‐neoplastic network that drives tumor cell proliferation, invasion, and metastasis [17, 18]. Furthermore, aberrant epigenetic regulation (such as dysregulated expression of specific microRNAs) and an immunosuppressive tumor microenvironment enriched in tumor‐associated macrophages promote malignant progression and immune evasion [19, 20].

Surgery remains the primary treatment for localized UPS, with the aim of achieving wide or radical excision and obtaining microscopically negative margins (R0 resection), as the presence of positive margins directly contributes to a significantly higher risk of local tumor recurrence [21, 22]. For metastatic or unresectable cases, chemotherapy remains the standard systemic treatment [10]. Currently, no UPS‐specific chemotherapy regimen has been established, and first‐line treatment for advanced STS continues to rely on anthracycline‐based protocols [12, 23]. The EORTC 62012 phase III trial demonstrated that single‐agent doxorubicin therapy in patients with advanced high‐grade STS yielded a median OS of 12.8 months, an objective response rate (ORR) of 14%, and a median progression‐free survival (PFS) of 4.6 months. Although combination therapy with ifosfamide increased the ORR to 26% and prolonged median PFS to 7.4 months, it did not confer a significant improvement in OS (median OS, 14.3 months, adjusted hazard ratio [HR] = 0.83, *p* = 0.076) [24]. However, anthracycline‐based chemotherapy is associated with substantial cardiotoxicity and hematological adverse effects, and in patients with prior intolerance to chemotherapy, as in the present case, the exploration of alternative systemic treatments is warranted [25].

With advances in oncology and immunology, targeted immunotherapeutic strategies have demonstrated promising applications [26]. In recent years, multiple targeted agents (such as anlotinib and pazopanib) have been employed in the treatment of advanced UPS [27, 28]. Anlotinib is a novel multi‐targeted tyrosine kinase inhibitor that simultaneously acts on multiple key pathways, including tumor proliferation and angiogenesis [28]. It effectively inhibits VEGF/VEGFR signaling by selectively targeting VEGFR‐2/3 and FGFR‐1 to 4 [29]. Furthermore, anlotinib inhibits the activity of PDGFRα/β, c‐Kit, Ret, Aurora‐B, c‐FMS, and discoidin domain receptor 1, thereby significantly suppressing tumor growth [29]. A Phase II study of anlotinib as second‐line therapy for advanced STS demonstrated an ORR of 5.3% in patients with UPS, a 12‐week PFS rate of 58%, a median PFS of 4.1 months, and a median OS of 11 months [28]. The patient presented with advanced UPS that was unsuitable for chemotherapy, a clinical scenario for which no established standard treatment regimen currently exists. Notably, the ALTER‐S003 study indicated that anlotinib as first‐line therapy in patients with advanced STS who were ineligible for chemotherapy achieved clinical benefit rates of 65.4% (17/26) at 4 months and 38.5% (10/26) at 6 months, with a favorable safety profile. Common grade 3 adverse events (AEs) included hypertension (17.2%), supporting the potential role of anlotinib as a first‐line therapeutic alternative for patients with advanced STS who are not candidates for chemotherapy [30].

Immune checkpoint inhibitors targeting programmed cell death protein‐1 (PD‐1) and its ligand programmed cell death ligand 1 (PD‐L1) antibodies have demonstrated efficacy against STS, with greater potential observed when combined with targeted therapies [31]. Sintilimab is a fully human IgG4 monoclonal antibody that blocks PD‐1 binding to PD‐L1 or PD‐L2 and exhibits a higher affinity for human PD‐1 than nivolumab and pembrolizumab [32]. Sintilimab is currently indicated for multiple solid tumors, including classical Hodgkin lymphoma, non‐small cell lung cancer, and hepatocellular carcinoma [33, 34, 35, 36]. Recently, the combination of sintilimab and anlotinib has shown promising prospects for the treatment of advanced sarcoma. Results from a multicenter retrospective study indicated that anti‐angiogenic agents (anlotinib or apatinib) combined with PD‐1 inhibitors (camrelizumab or sintilimab) achieved a six‐month progression‐free survival rate (PFSR) of 51.3%, with an ORR of 20.5%, median PFS of 7.0 months, and median OS of 17.2 months [37]. Another prospective, single‐arm, phase II trial evaluating sintilimab plus anlotinib in previously treated patients with advanced sarcoma reported an ORR of 30.9% (95% CI, 16.4%–45.6%), a median PFS of 5.0 months (95% CI, 2.8–10.2), and a 1‐year OS rate of 62.6%. The combination regimen was well‐tolerated, with hypertension and hyponatremia being the most common grade ≥ 3 AEs, each reported in 4.8% of the patients [38]. The advantage of combination therapy lies in the dual action of anlotinib; it inhibits tumor vascular growth while modulating the tumor microenvironment to reverse immunosuppression, thereby enhancing the tumor‐targeting capacity of sintilimab‐activated immune cells [39, 40]. Studies have confirmed that this combination increases the proportion of CD4^+^ T cells, CD8^+^ T cells, and natural killer (NK) cells within tumors [39, 41]. Although several studies have reported the efficacy of sintilimab combined with anlotinib in advanced sarcoma, evidence remains limited for elderly patients with UPS and lung metastases who are unable to tolerate chemotherapy. This case report describes the use of this combination therapy in an elderly patient with positive surgical margins, intolerance to first‐line chemotherapy, and subsequent local recurrence with lung metastasis, who subsequently achieved a durable PR in the pulmonary lesions.

Following treatment with sintilimab plus anlotinib, the patient demonstrated a reduction in pulmonary lesions by the second treatment cycle, with continued tumor shrinkage observed after five cycles. A follow‐up review on December 17, 2025, indicated a marked PR of the pulmonary lesions, representing a 76.62% reduction from the baseline. At the time of writing, the patient had achieved six months of PFS and remains under follow‐up. Given that this is a single‐case report, the findings should be interpreted with caution. The combination of sintilimab and anlotinib appeared to demonstrate clinical efficacy in this patient; however, larger prospective studies are needed to further evaluate its efficacy and safety in UPS patients who are ineligible for chemotherapy. This report has several limitations. First, it describes a single patient without a control group, which limits the generalizability of the findings. Second, as immunohistochemical testing was not performed on the amputated specimens, the diagnosis of local recurrence was based primarily on the morphological features of the primary tumor and clinical imaging findings. Third, response assessment was conducted without central radiological review. Fourth, the follow‐up period was relatively short (6 months), limiting the assessment of long‐term response and toxicity.

This report presents the case of a patient with UPS and reviews recent diagnostic and therapeutic advances for this rare tumor. For patients with advanced disease who are ineligible for chemotherapy, conventional systemic treatment options remain limited. Owing to the scarcity of advanced UPS cases, large‐scale clinical trials and the establishment of standardized treatment protocols are extremely challenging, leaving the management of this patient population at an exploratory stage. In the present case, combination therapy with a PD‐1 monoclonal antibody and a tyrosine kinase inhibitor resulted in favorable therapeutic outcomes, suggesting that this combined strategy warrants further investigation. Future large‐scale prospective studies are needed to clarify and optimize systemic treatment regimens for UPS.

## Author Contributions

**Yan Chen:** data curation, investigation, validation, formal analysis, writing – original draft. **Ronghua Chu:** conceptualization, methodology, supervision, project administration, writing – review and editing.

## Funding

The authors have nothing to report.

## Ethics Statement

This study did not require approval by an ethics committee, since all patient information presented in this case report was anonymized.

## Consent

Written informed consent was obtained from the patient for the publication of this case report and associated images.

## Conflicts of Interest

The authors declare no conflicts of interest.

## Data Availability

The data that support the findings of this study are available from the corresponding author upon reasonable request.
